# Supplementary material for: Blood-based epigenetic instability linked to human aging and disease
Source: Nat Commun. 2026 Feb 14;17:2754. doi: 10.1038/s41467-026-69430-z (PMC13018287; doi:10.1038/s41467-026-69430-z)
Supplement: Supplementary file 1 — Supplementary Information [file 41467_2026_69430_MOESM1_ESM.pdf]

Blood-Based Epigenetic Instability Linked to Human Aging and Disease

Salman Basrai[1,2], Ido Nofech-Mozes[1,2], Rajesh Detroja[3], Fernando L. Scolari[4,5], Mehran Bakhtiari[3], Andrea Arruda[3], Tracy Murphy[6], Scott V. Bratman[3,7], Steven M. Chan[3,8], Mark D. Minden[3,6,8], Jae-Sook Ahn[9], Dennis D. H. Kim[3,6], Robert Kridel[3,8], Filio Billia[4,10], Sagi Abelson[1,2]*

[1] Ontario Institute for Cancer Research, Toronto, ON, Canada.
[2] Department of Molecular Genetics, University of Toronto, Toronto, ON, Canada.
[3] Princess Margaret Cancer Centre, University Health Network, Toronto, ON, Canada.
[4] Peter Munk Cardiac Centre, University Health Network, Toronto, ON, Canada.
[5] Hospital de Clínicas de Porto Alegre, Porto Alegre, Rio Grande do Sul, Brazil.
[6] Division of Medical Oncology and Hematology, Princess Margaret Cancer Centre, Toronto, ON, Canada.
[7] Department of Radiation Oncology, University of Toronto, Toronto, ON, Canada.
[8] Department of Medical Biophysics, University of Toronto, Toronto, ON, Canada.
[9] Chonnam National University Hwasun Hospital, Chonnam National University, Hwasun, Korea.
[10] Department of Physiology, University of Toronto, Toronto, ON, Canada.

* Corresponding author: SAbelson@oicr.on.ca

Table of contents

- **Supplementary Figures**
- **Supplementary Note**
- **Supplementary References**

Supplementary Figures

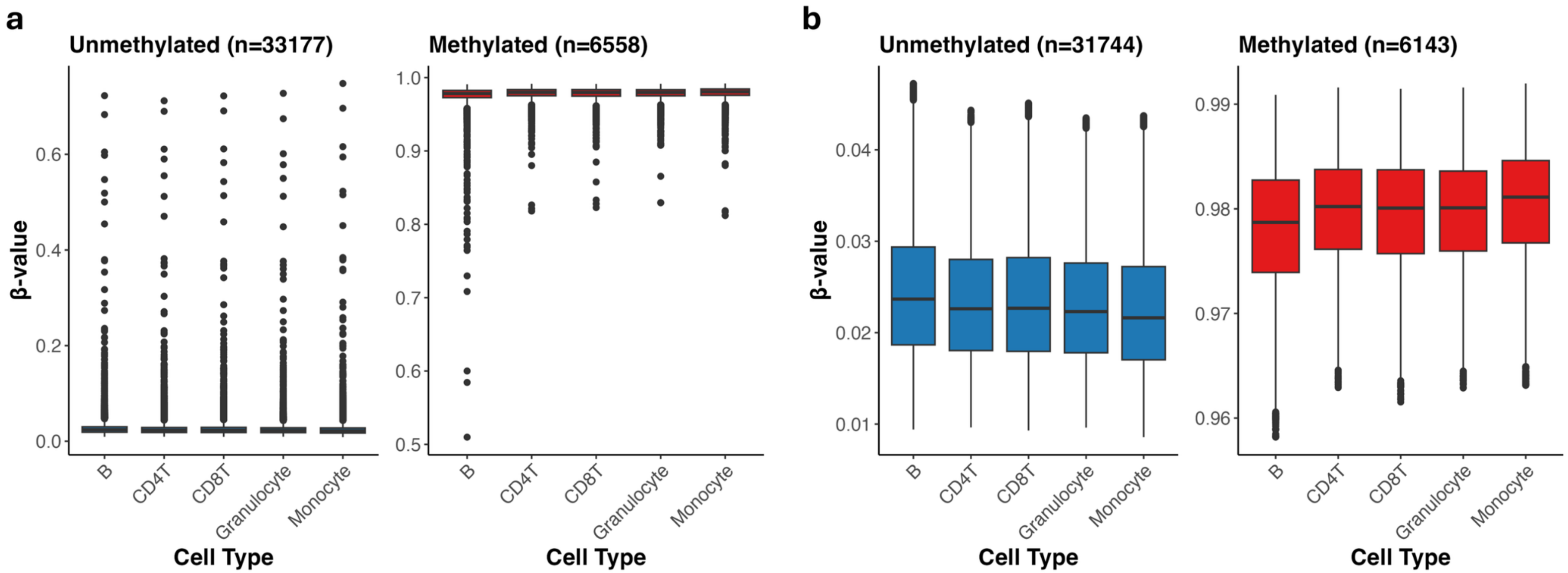

Supplementary Figure 1. Blood cell proportion-based filtering. β-values of the 10% lowest-variance CpG sites identified in a large cohort of young, healthy individuals (n = 1,658; age = 18) were analyzed across five purified blood cell types. **a**, Average methylation levels of highly stable CpG sites across purified blood cell populations (Hannon et al., 2021)[1]. CpGs with outlier β-values are shown as dots and were excluded from further analysis. **b**, Final set of 31,744 unmethylated and 6,143 methylated ESLs remaining after filtering.

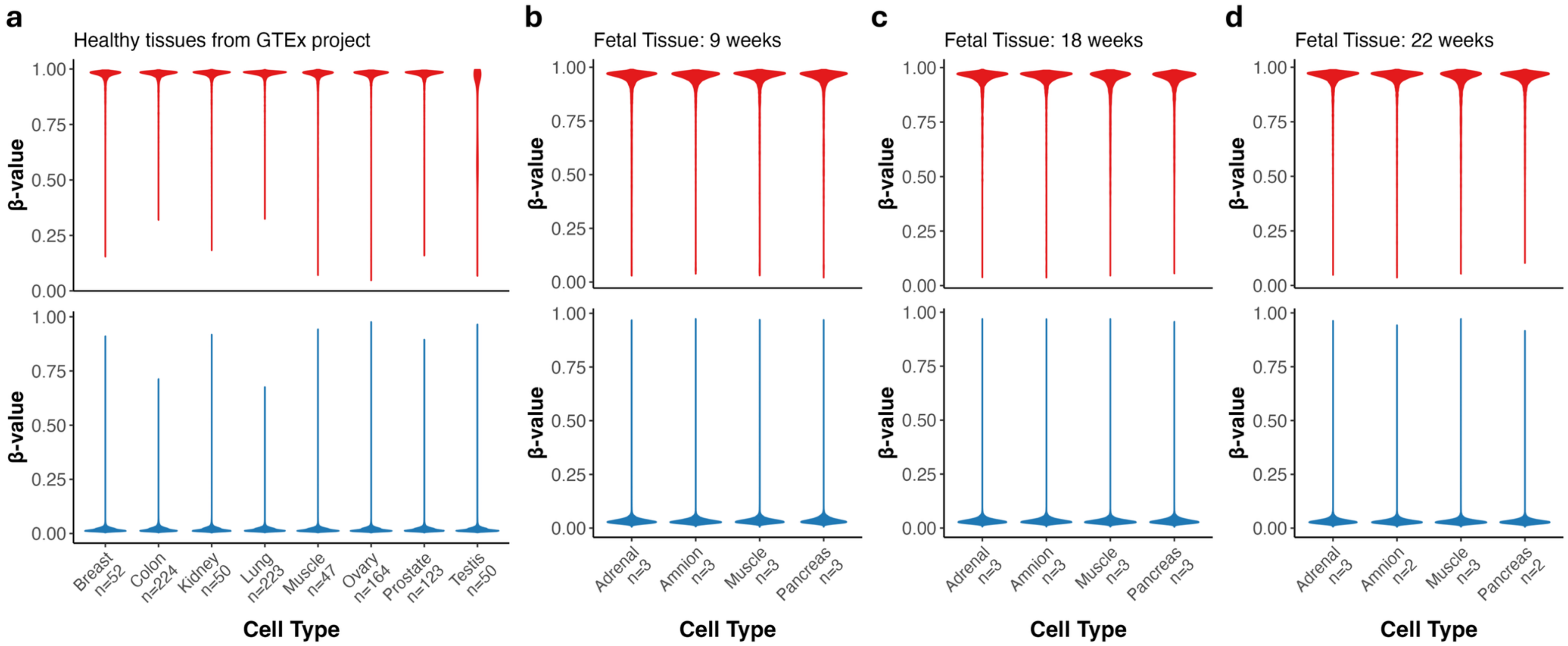

Supplementary Figure 2. Stability of blood-derived epigenetically stable loci (ESLs) across human tissues and developmental stages. **a**, Violin plots showing β-values of hypermethylated (top) and hypomethylated (bottom) ESLs in healthy adult tissues from the GTEx project, assayed using the Illumina EPIC array. **b-d**, Violin plots showing β-values of ESLs in four fetal tissues (adrenal, amnion, muscle, and pancreas) during the first and second trimesters of gestation, measured with the Illumina 450K array. Each violin represents the average β-values across all samples within the corresponding tissue.

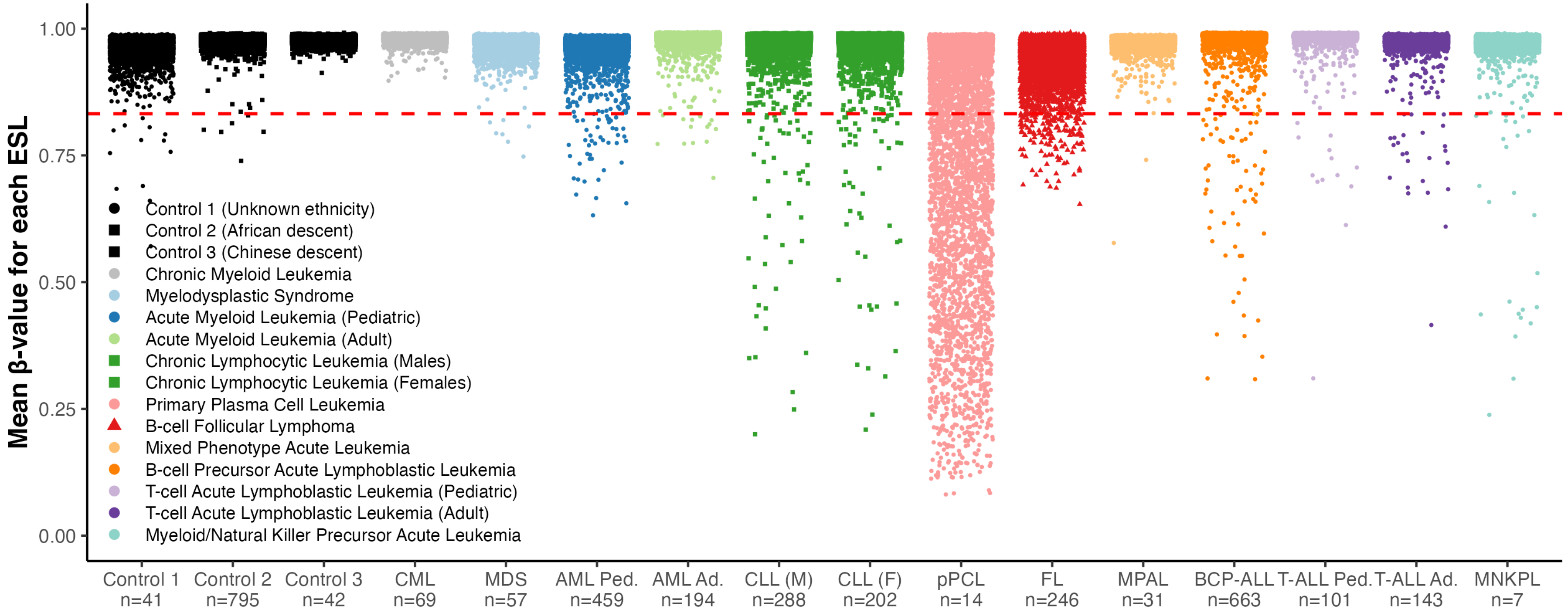

Supplementary Figure 3. Destabilization of methylated ESLs in hematologic malignancies. Average β-values of 6,143 ESLs that are typically methylated in healthy individuals (β-values near 1) are shown across control and cancer cohorts. Each point represents the average β-value for a single ESL in a given cohort. The dashed red line indicates the destabilization threshold established from the control cohorts (β-value = 0.8321). Marker shapes denote tissue source: circles = bone marrow, squares = peripheral blood, triangles = lymphoid tissue. Abbreviations: CML – Chronic Myeloid Leukemia; MDS – Myelodysplastic Syndromes; AML – Acute Myeloid Leukemia; CLL – Chronic Lymphocytic Leukemia; pPCL – Primary Plasma Cell Leukemia; FL – Follicular Lymphoma; MPAL – Mixed Phenotype Acute Leukemia; BCP-ALL – B-cell Precursor Acute Lymphoblastic Leukemia; T-ALL – T-cell Acute Lymphoblastic Leukemia; MNKPL – Myeloid/Natural Killer Cell Precursor Acute Leukemia; Ped – Pediatric; Ad – Adult; M – Male; F – Female.

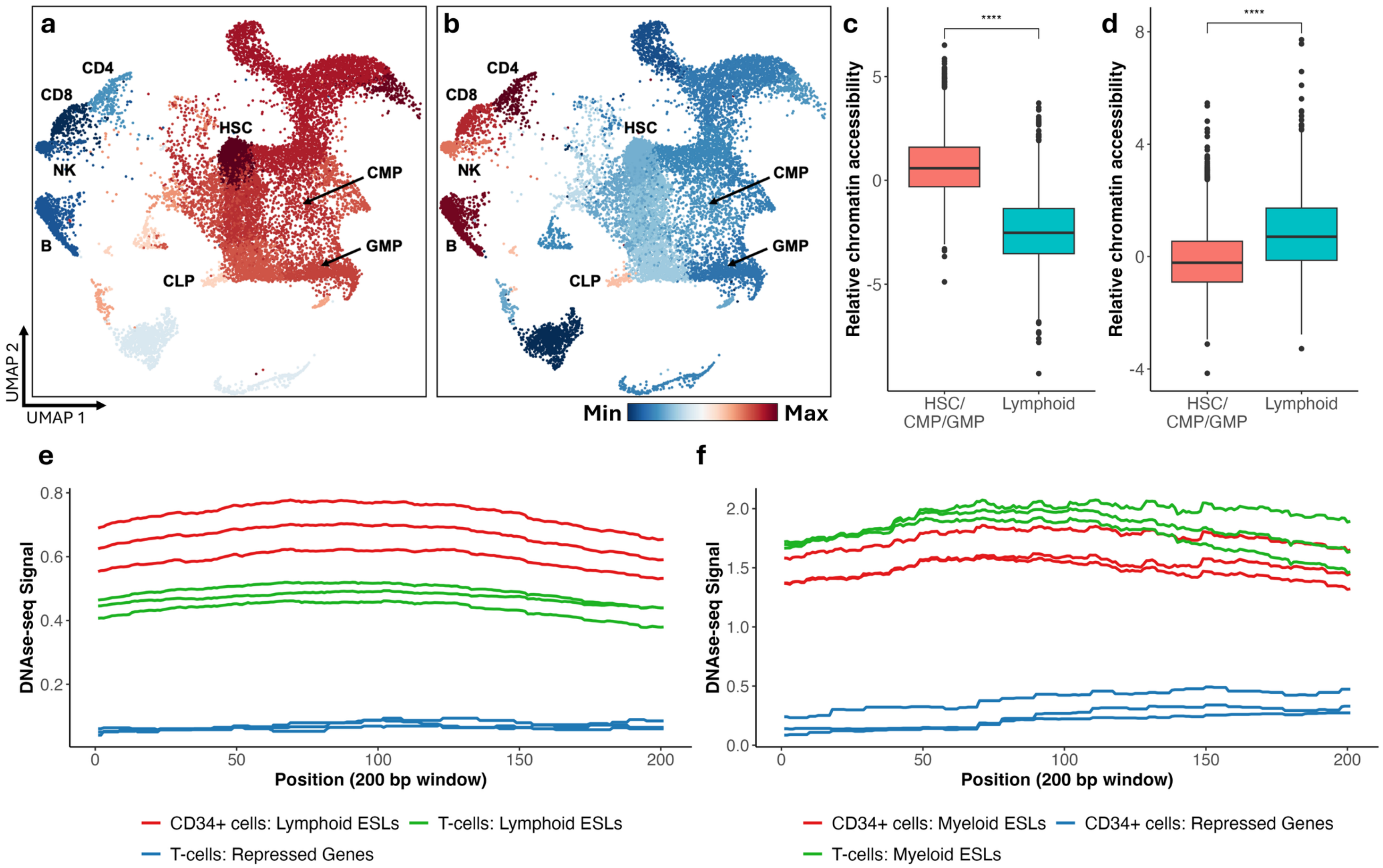

Supplementary Figure 4. Chromatin accessibility patterns in non-malignant hematopoietic cells at lineage-enriched perturbed ESLs. a-b, UMAP projections of single-cell ATAC-seq profiles from non-malignant hematopoietic cells (n = 6,088) isolated from patients with myelofibrosis (Izzo F, et al. 2024)[2]. JAK2^{V617F}-positive cells were omitted from this analysis. Cells are colored by their relative chromatin accessibility at genomic regions overlapping **a,** lymphoid-enriched or **b**, myeloid-enriched ESLs, based on ChromVAR deviation scores[3] reflecting enrichment relative to a background model. Red indicates higher relative accessibility (less compacted chromatin); blue indicates

lower relative accessibility (more compacted chromatin). **c-d**, Relative chromatin accessibility at ESL-associated regions in hematopoietic stem and progenitor cells (HSCs, CMPs, GMPs) compared with lymphoid cell types (CLP, B, $CD4^+$ T, $CD8^+$ T, NK), for **c**, lymphoid-enriched ESLs and **d**, myeloid-enriched ESLs. $^{****}P < 2.2 \times 10^{-16}$ by two-sided Wilcoxon test. **e**, DNase-seq data from three T-cell samples (ENCODE IDs: ENCSR414IHC, ENCSR366YTD, and ENCSR040DGJ) and three $CD34^+$ cell samples (ENCODE IDs: ENCSR495INQ, ENCSR453EVC, and ENCSR122VUW) were analyzed. 200 bp windows centered on each lymphoid-enriched ESL (n = 1,075) were used to calculate the average DNase-seq signal per base across T-cell (green) and $CD34^+$ cells (red). For comparison, the average signal across T-cell samples was also computed for the promoters (200 bp upstream of the transcription start site) of seven genes known to be largely repressed in T cells (KLF1, SPI1, ALB, AFP, MYH7, RBFOX3, CAMK2A; blue). **f**, A similar analysis was performed for myeloid-enriched ESLs (n = 80) and promoters of seven genes repressed in $CD34^+$ cells (GATA1, KLF1, PAX5, EBF1, FBP1, FBP2, NOS3).

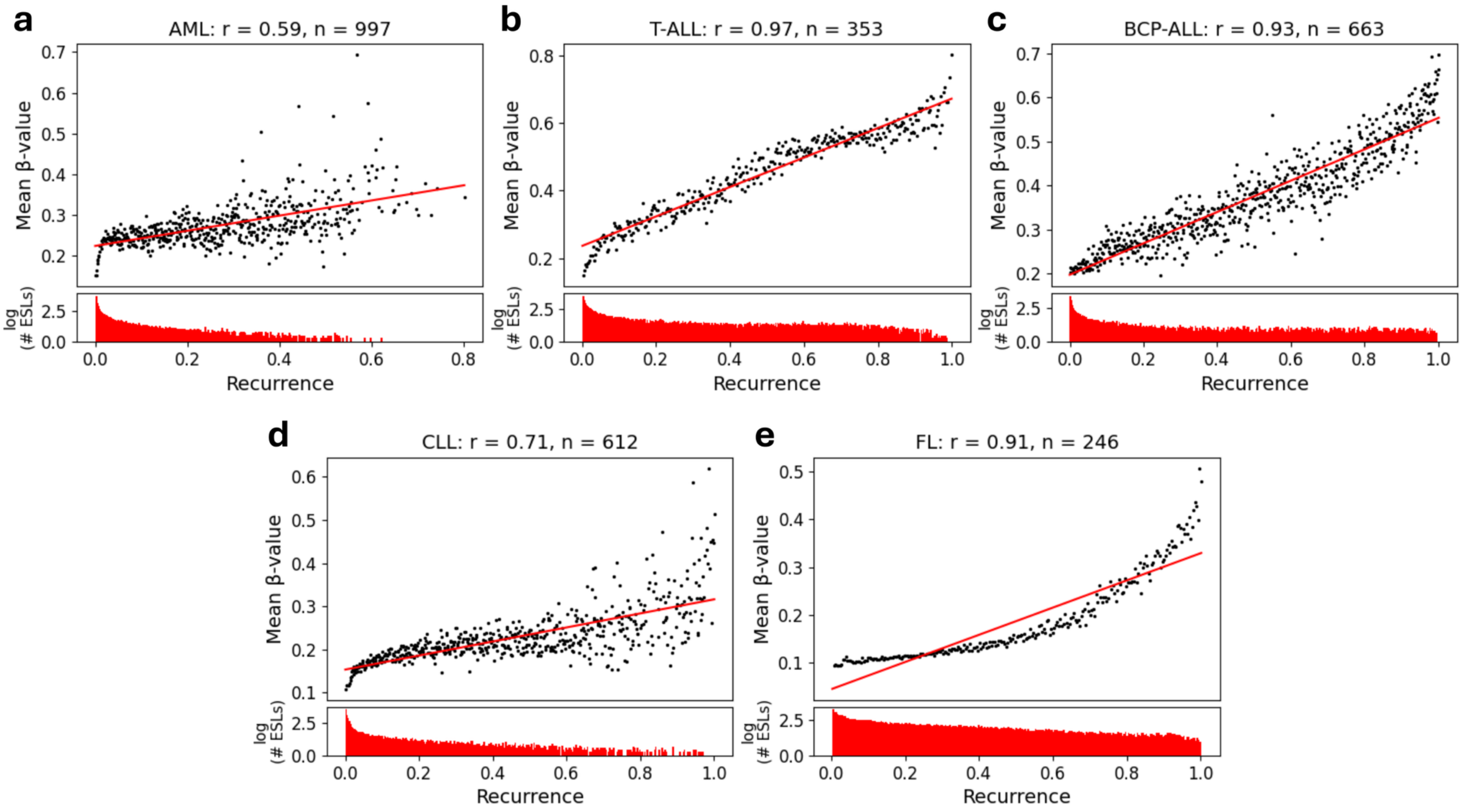

Supplementary Figure 5. Correlation between recurrence and β-value across individual cancer cohorts. Recurrence of ESLs is plotted on the x-axis as the proportion of patients in which a given locus exceeds the destabilization threshold. The y-axis represents the mean β-value calculated only across patients in whom that locus is perturbed. Thus, the plot reflects the relationship between the frequency of ESL destabilization across individuals and the average methylation magnitude among those affected samples. Cancer type, sample size, and Pearson's correlation coefficient (r) are indicated in each panel. $P < .001$ for all analyses. Bottom panels display the number of ESLs ($\log_{10}$ scale) at different recurrence levels.

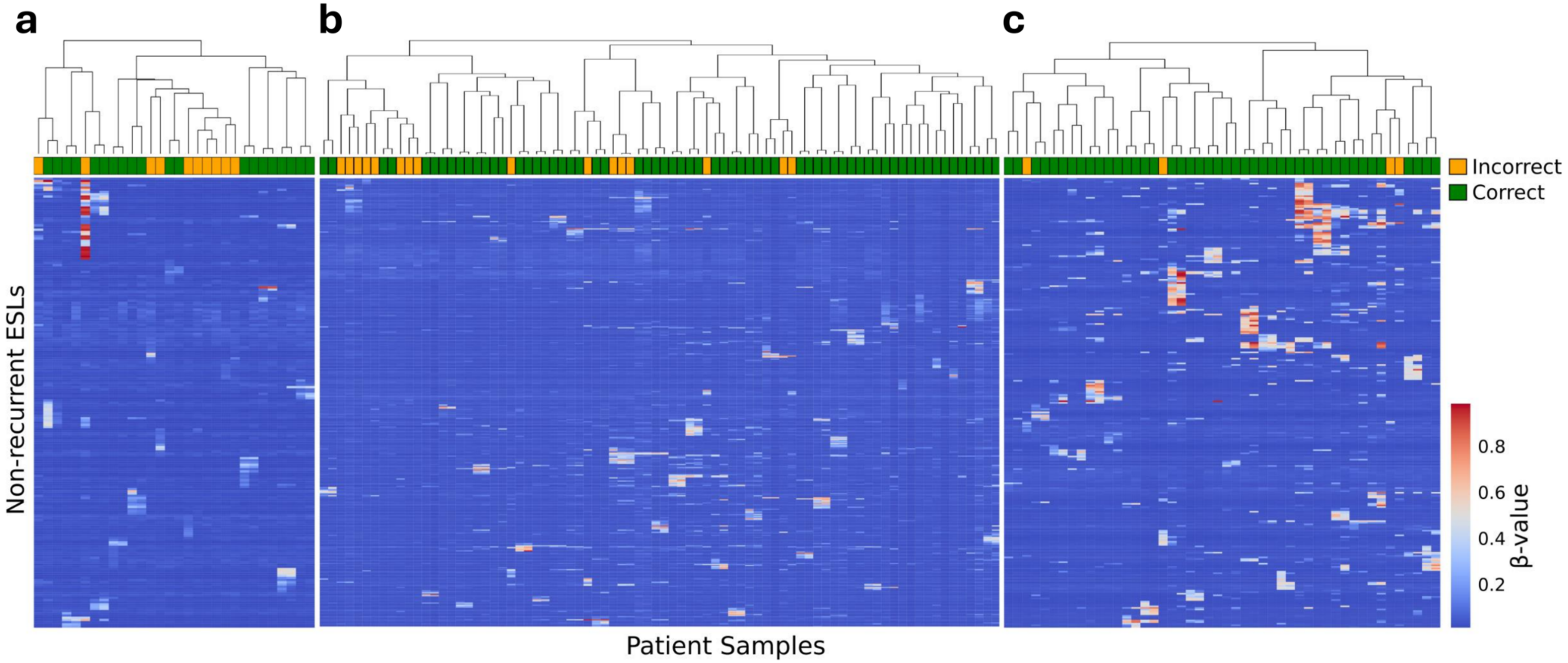

Supplementary Figure 6. Low-recurrence ESLs identified at diagnosis enable accurate pairing of diagnosis and relapse samples. Hierarchical clustering of paired diagnosis and relapse samples using the 15 low recurrence ESLs with the highest methylation, selected from each patient's diagnosis sample. **a**, AML (n = 15), **b**, CLL (n = 40), and **c**, BCP-ALL (n = 24). Correct diagnosis–relapse pairings are indicated in green; incorrect pairings are shown in orange. Permutation test: $P = 0.001$ for all analyses.

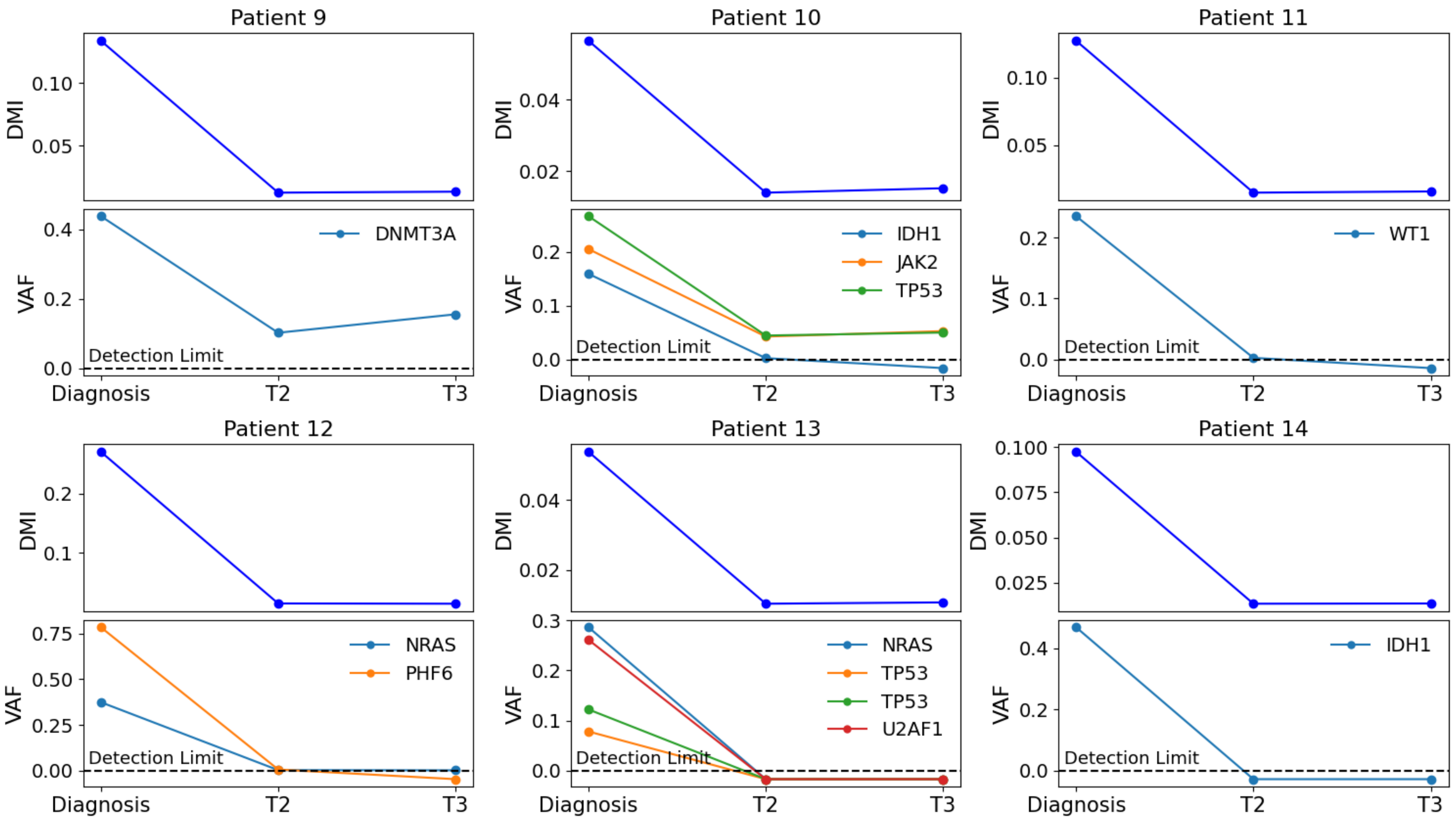

Supplementary Figure 7. Longitudinal analysis of peripheral blood samples from six additional AML patients. Samples were collected at diagnosis and at two subsequent post-therapy time points. Top panels show DMI levels; bottom panels track the variant allele fractions (VAFs) of leukemic mutations identified at diagnosis.

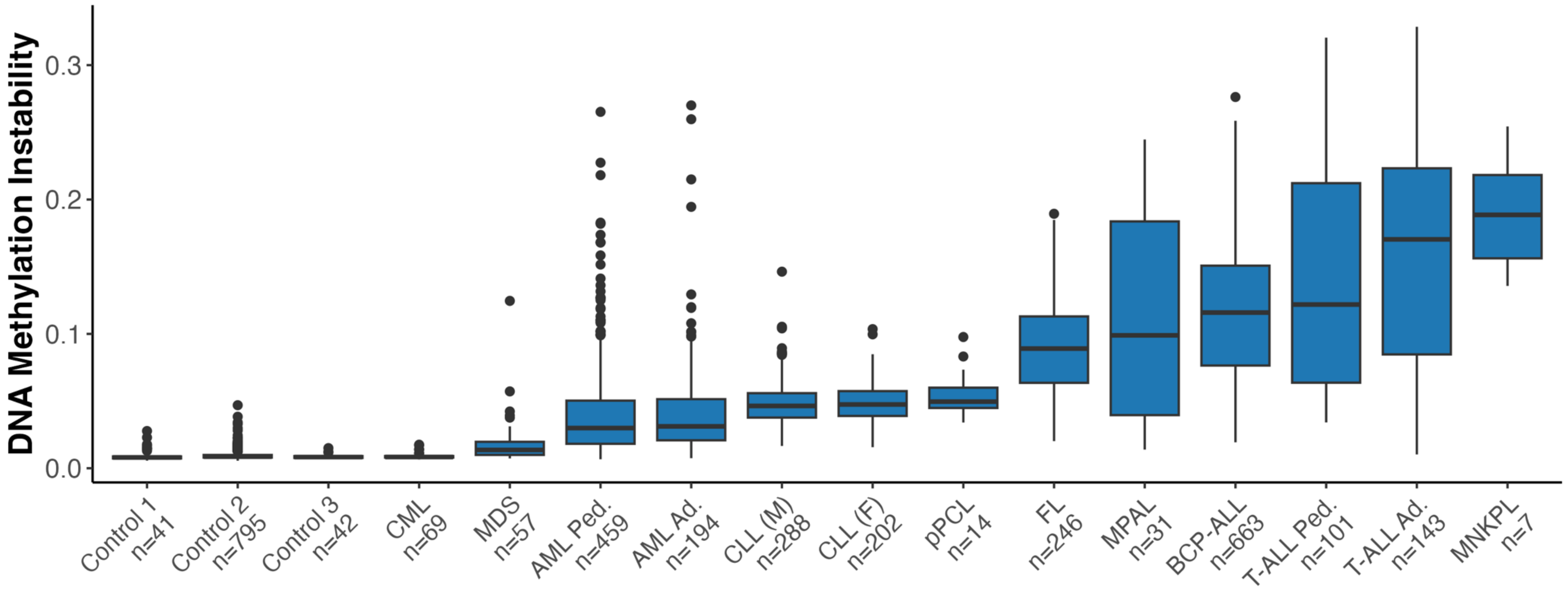

Supplementary Figure 8. DMI values in healthy controls and patients with hematological malignancies. Boxplot of DNA methylation instability (DMI) values corresponding to all control and cancer samples depicted in Fig. 2. DMI was calculated as the standard deviation of β-values for all ESLs with recurrence greater than 5%.

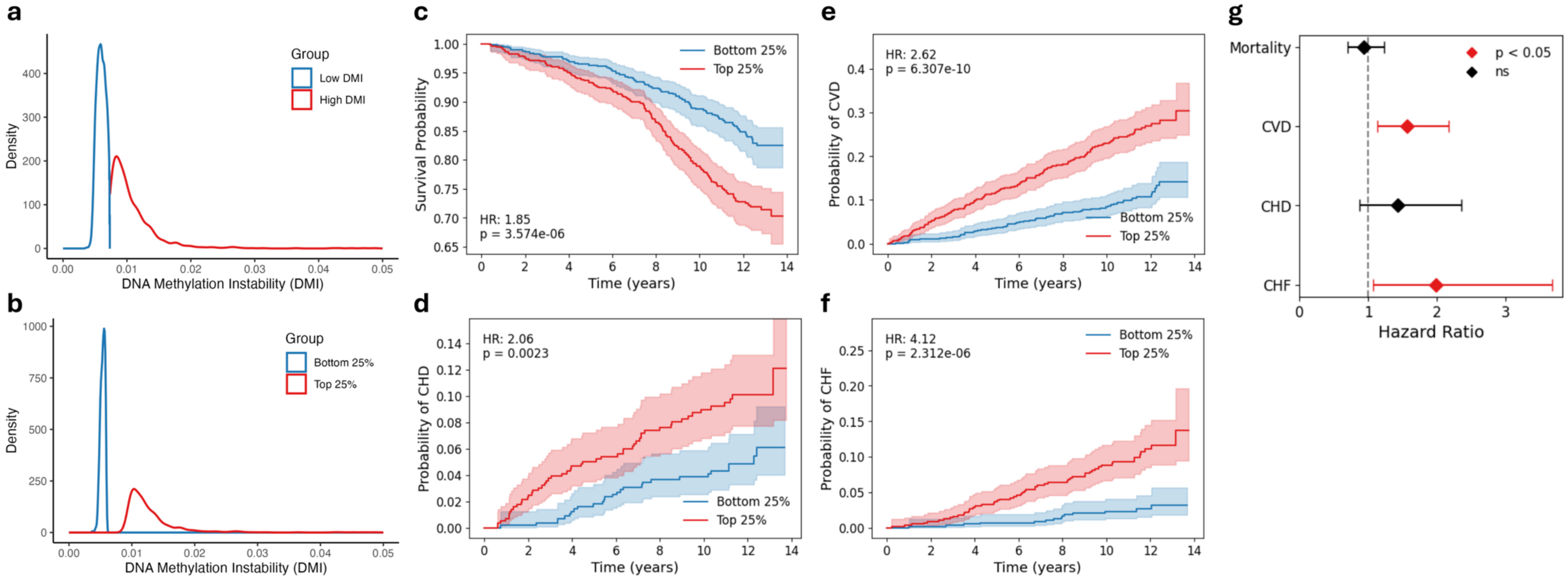

Supplementary Figure 9. Association between DMI and cardiovascular complications in individuals with extreme DMI values. a, Distribution of DMI values in participants from the Framingham Heart Study (Exam 8) designated as "low DMI" or "high DMI" based on a median split (n = 2,281). **b**, Distribution of DMI values in the top and bottom quartiles of the cohort (n = 1,141). **c-f**, Univariate Kaplan-Meier analyses of the top and bottom quartiles for **c**, mortality, **d**, coronary heart disease (CHD), **e**, cardiovascular disease (CVD), and **f**, congestive heart failure (CHF). **g**, Forest plot showing the effect of DMI (bottom quartile vs. top quartile) on each endpoint using multivariate Cox proportional hazards regression, adjusted for age and biological sex. Mortality P=0.624; CVD P=0.007; CHD P=0.158; CHF P=0.031.

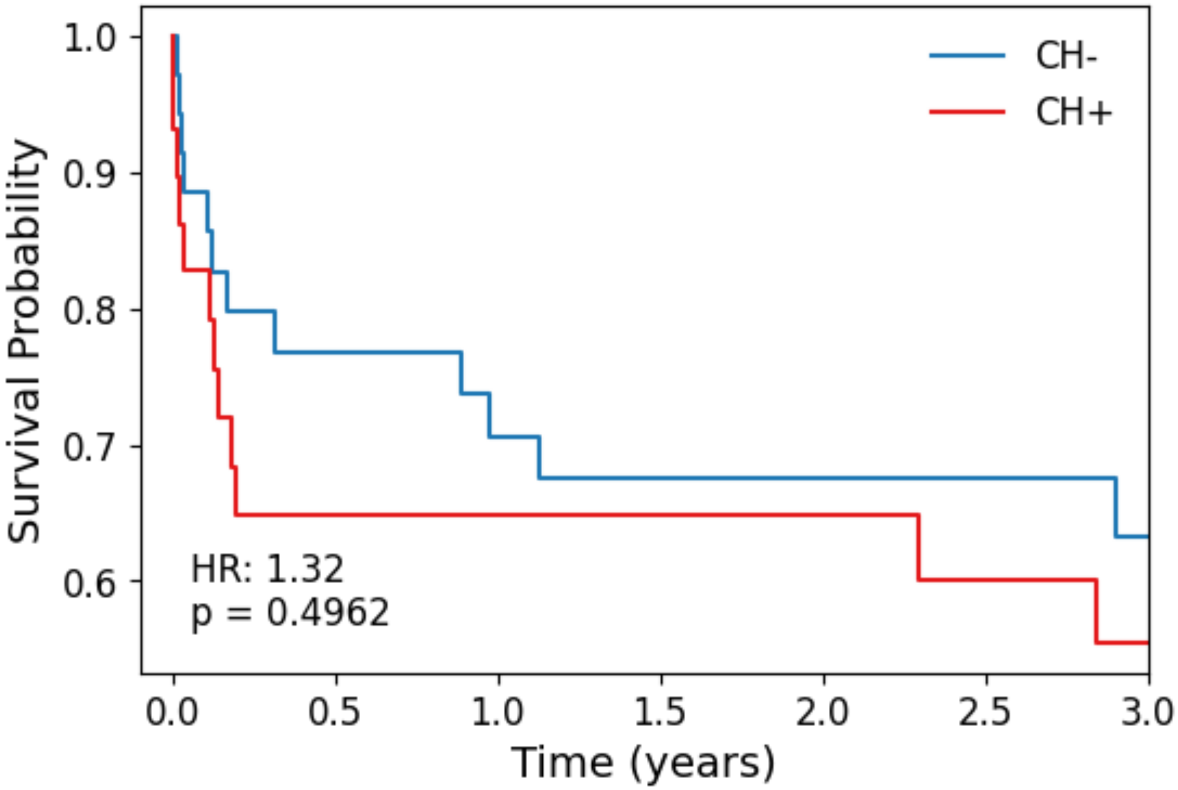

Supplementary Figure 10. Survival analysis of the cardiogenic shock sub-cohort based on clonal hematopoiesis status alone. Univariate Kaplan-Meier survival analysis of 64 cardiogenic shock patients, stratified by the presence or absence of clonal hematopoiesis mutations. The hazard ratio (HR) and *P* value are indicated.

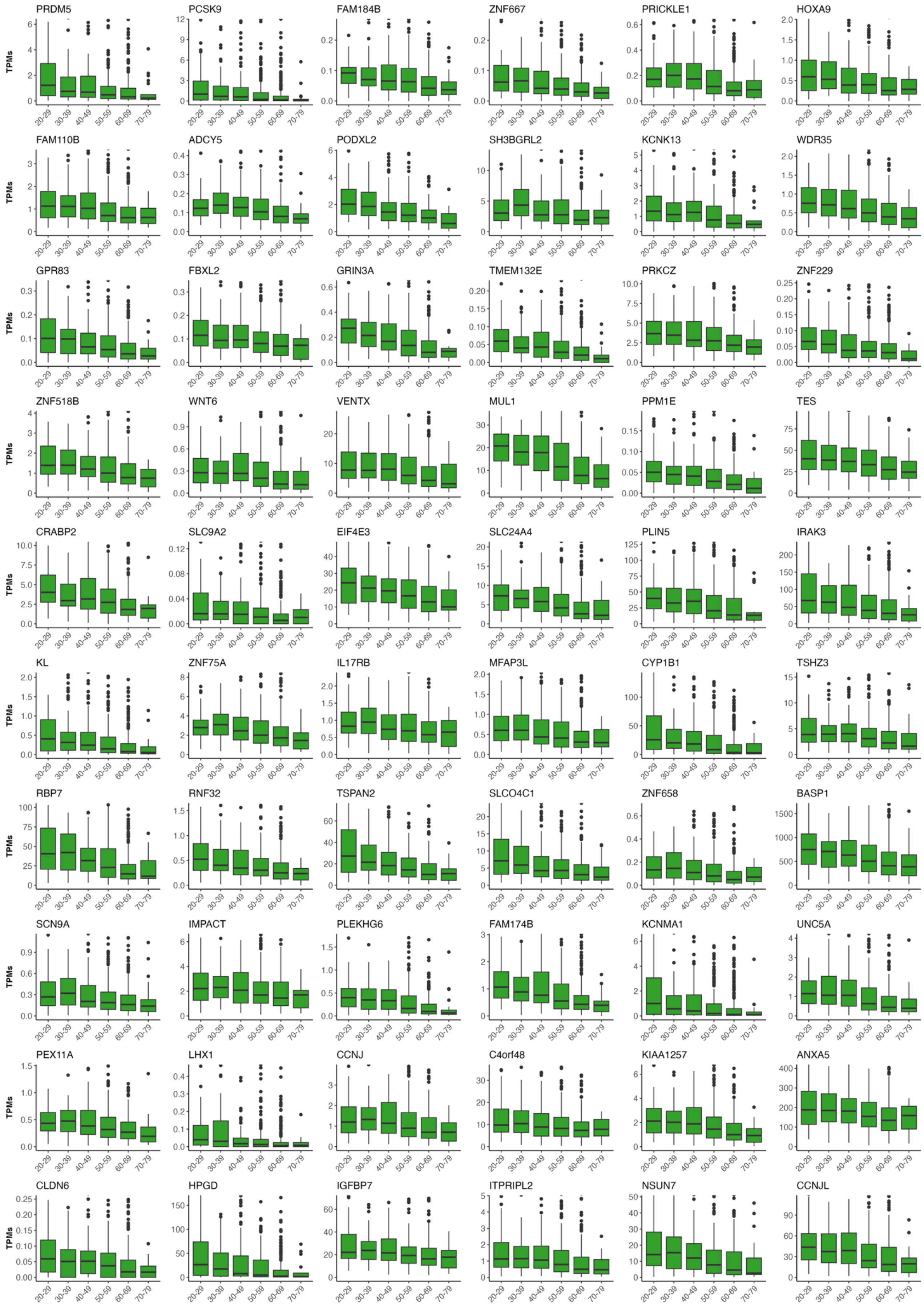
PRDM5
PCSK9
FAM184B
ZNF667
PRICKLE1
HOXA9
FAM110B
ADCY5
PODXL2
SH3BGRL2
KCNK13
WDR35
GPR83
FBXL2
GRIN3A
TMEM132E
PRKCZ
ZNF229
ZNF518B
WNT6
VENTX
MUL1
PPM1E
TES
CRABP2
SLC9A2
EIF4E3
SLC24A4
PLIN5
IRAK3
KL
ZNF75A
IL17RB
MFAP3L
CYP1B1
TSHZ3
RBP7
RNF32
TSPAN2
SLCO4C1
ZNF658
BASP1
SCN9A
IMPACT
PLEKHG6
FAM174B
KCNMA1
UNC5A
PEX11A
LHX1
CCNJ
C4orf48
KIAA1257
ANXA5
CLDN6
HPGD
IGFBP7
ITPRIPL2
NSUN7
CCNJL
TPMs
20-29
30-39
40-49
50-59
60-69
70-79

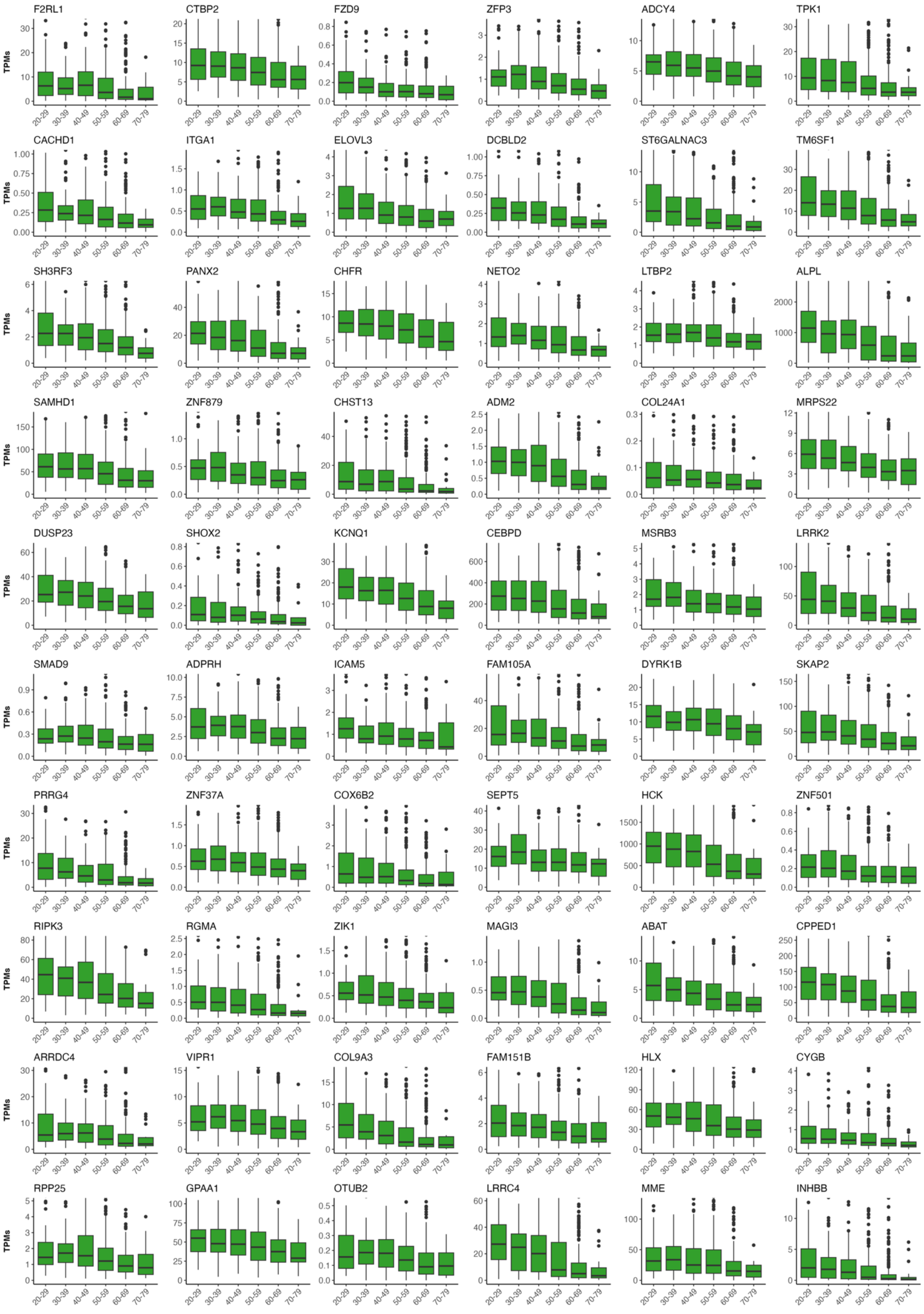
F2RL1
CTBP2
FZD9
ZFP3
ADCY4
TPK1
CACHD1
ITGA1
ELOVL3
DCBLD2
ST6GALNAC3
TM6SF1
SH3RF3
PANX2
CHFR
NETO2
LTBP2
ALPL
SAMHD1
ZNF879
CHST13
ADM2
COL24A1
MRPS22
DUSP23
SHOX2
KCNQ1
CEBPD
MSRB3
LRRK2
SMAD9
ADPRH
ICAM5
FAM105A
DYRK1B
SKAP2
PRRG4
ZNF37A
COX6B2
SEPT5
HCK
ZNF501
RIPK3
RGMA
ZIK1
MAGI3
ABAT
CPPED1
ARRDC4
VIPR1
COL9A3
FAM151B
HLX
CYGB
RPP25
GPAA1
OTUB2
LRRC4
MME
INHBB
TPMs
20-29
30-39
40-49
50-59
60-69
70-79

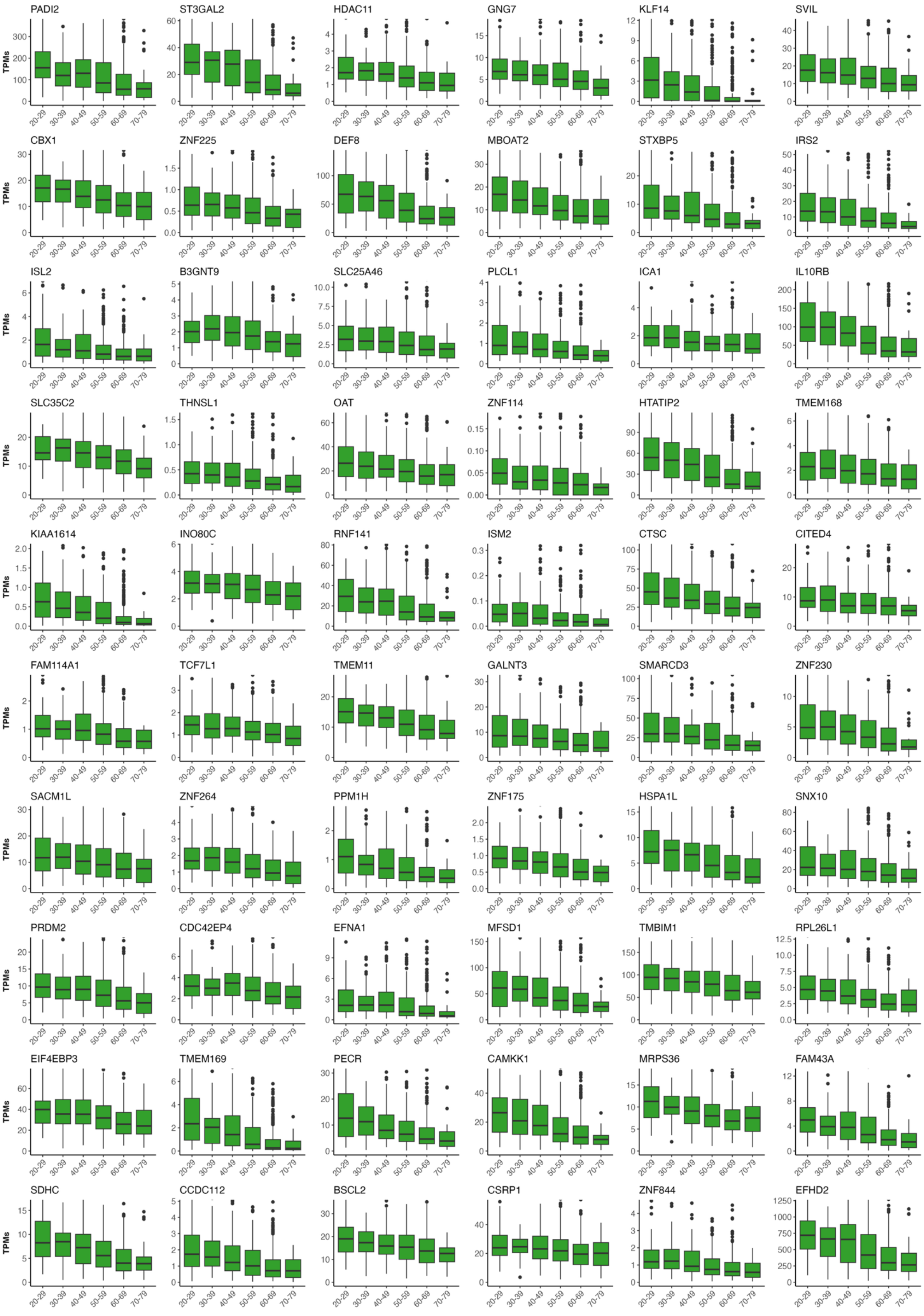
PADI2
ST3GAL2
HDAC11
GNG7
KLF14
SVIL
CBX1
ZNF225
DEF8
MBOAT2
STXBP5
IRS2
ISL2
B3GNT9
SLC25A46
PLCL1
ICA1
IL10RB
SLC35C2
THNSL1
OAT
ZNF114
HTATIP2
TMEM168
KIAA1614
INO80C
RNF141
ISM2
CTSC
CITED4
FAM114A1
TCF7L1
TMEM11
GALNT3
SMARCD3
ZNF230
SACM1L
ZNF264
PPM1H
ZNF175
HSPA1L
SNX10
PRDM2
CDC42EP4
EFNA1
MFSD1
TMBIM1
RPL26L1
EIF4EBP3
TMEM169
PECR
CAMKK1
MRPS36
FAM43A
SDHC
CCDC112
BSCL2
CSRP1
ZNF844
EFHD2
TPMs
20-29
30-39
40-49
50-59
60-69
70-79

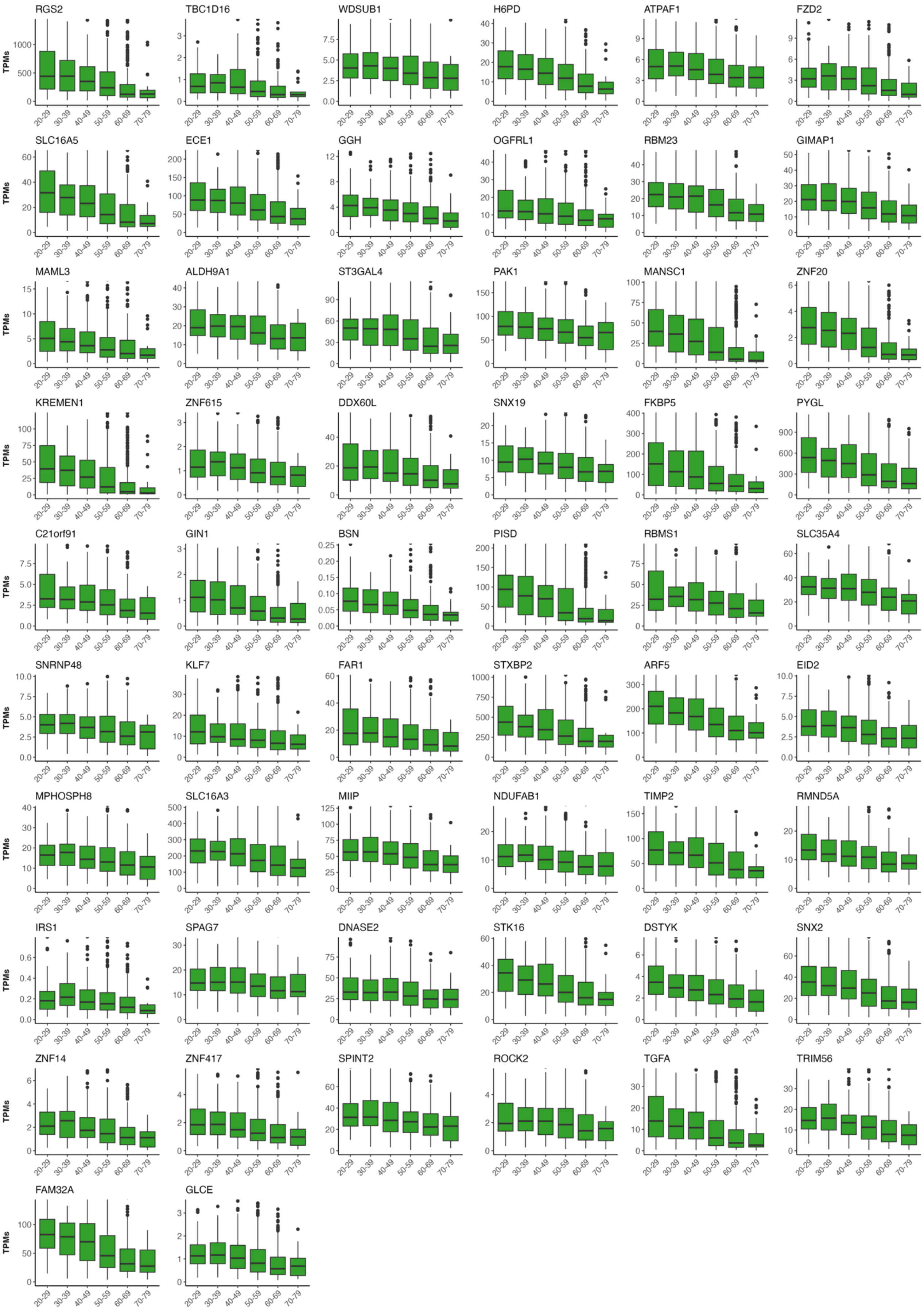
RGS2
TBC1D16
WDSUB1
H6PD
ATPAF1
FZD2
SLC16A5
ECE1
GGH
OGFRL1
RBM23
GIMAP1
MAML3
ALDH9A1
ST3GAL4
PAK1
MANSC1
ZNF20
KREMEN1
ZNF615
DDX60L
SNX19
FKBP5
PYGL
C21orf91
GIN1
BSN
PISD
RBMS1
SLC35A4
SNRNP48
KLF7
FAR1
STXBP2
ARF5
EID2
MPHOSPH8
SLC16A3
MIIP
NDUFAB1
TIMP2
RMND5A
IRS1
SPAG7
DNASE2
STK16
DSTYK
SNX2
ZNF14
ZNF417
SPINT2
ROCK2
TGFA
TRIM56
FAM32A
GLCE
TPMs
20-29
30-39
40-49
50-59
60-69
70-79

Supplementary Figure 11. Age-associated expression of genes with ESL-linked promoter methylation. Expression of 236 genes in peripheral blood from healthy donors, whose promoter regions contain ESLs with methylation levels inversely correlated with gene expression. Individuals were grouped into 10-year age bins using data from the Genotype-Tissue Expression (GTEx) Project[4] (n = 755). The y-axis shows transcript levels (TPM), and the x-axis indicates age groups: 20–29, 30–39, 40–49, 50–59, 60–69, and 70–79.

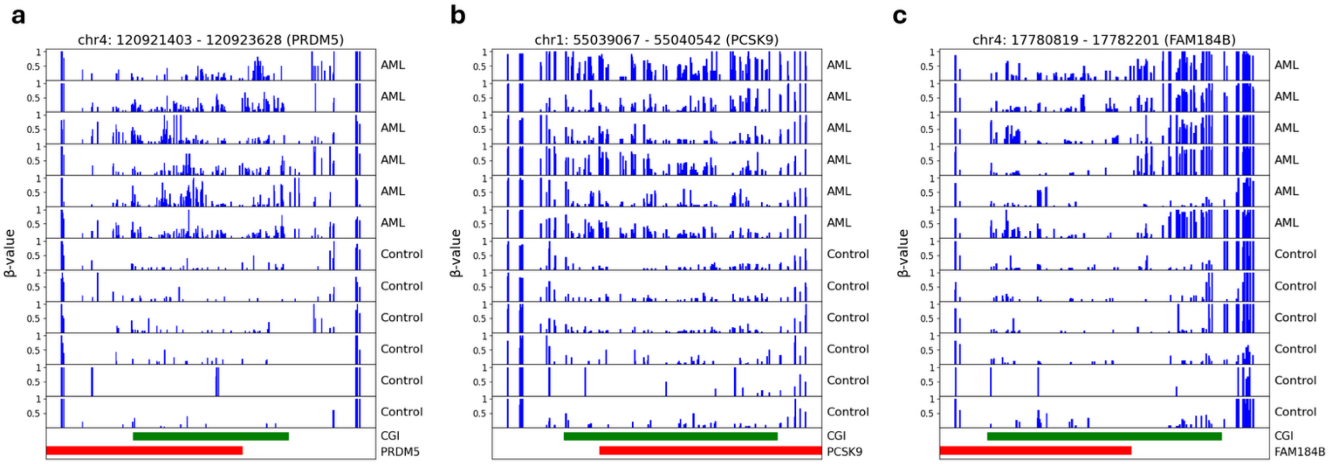

Supplementary Figure 12. Hypermethylation of promoter regions in AML. DNA methylation profiles across the promoter regions of **a**, *PRDM5*, **b**, *PCSK9*, and **c**, *FAM184B* in AML patients (n = 6) and healthy controls (n = 6). Methylation levels are displayed as β-values (proportion of methylated reads) and were obtained from the dataset reported by Wilson et al. (2022)[5], based on whole-genome bisulfite sequencing. Each row represents an individual sample, with AML cases and controls labeled. The gene bodies and associated CpG islands (CGI) are annotated in red and green, respectively.

Supplementary Note 1

<u>Statistical Analysis of Clustering Results</u>

Given a situation where we need to match 24 relapse BCP-ALL samples to their corresponding diagnosis samples (a total of 48 samples), we can calculate the probability of randomly making all the correct matches. The problem can be solved with combinatorics, where we are forming pairs from a set of items. The total number of possible pairings can be computed using the following formula where n is the number of pairs. For our case, $n = 24$.

Total Pairings = $(2n)! / (2^n * n!)$
Thus, the total number of ways to form pairs from 48 samples is given by:
Total Pairings = $(48)! / (2^{24} * 24!)$

Considering that there is only one correct pairing, where each diagnosis sample is matched with its corresponding relapse sample. The probability of randomly matching all samples correctly is given by the ratio of the correct pairing to the total number of possible pairings:

Probability = $1 / [(48)! / (2^{24} * 24!)]$
This simplifies to:
Probability = $(2^{24} * 24!) / (48!) = 8.385265\times10^{-31}$

The probability of correctly matching all 24 relapse samples to their corresponding diagnosis samples by random pairing is extremely close to zero.

Supplementary References

1. Hannon, E., Mansell, G., Walker, E., Nabais, M. F., Burrage, J., Kepa, A., … Mill, J. (2021). Assessing the co-variability of DNA methylation across peripheral cells and tissues: Implications for the interpretation of findings in epigenetic epidemiology. *PLoS Genetics*, *17*(3), e1009443.
2. Izzo F, Myers RM, Ganesan S, et al. Mapping genotypes to chromatin accessibility profiles in single cells. *Nature*. 2024;629(8014):1149–1157.
3. Schep AN, Wu B, Buenrostro JD, Greenleaf WJ. chromVAR: inferring transcription-factor-associated accessibility from single-cell epigenomic data. *Nat Methods*. 2017;14(10):975–978.
4. GTEx Consortium. The Genotype-Tissue Expression (GTEx) project. *Nat Genet*. 2013;45(6):580–585.
5. Wilson ER, Helton NM, Heath SE, et al. Focal disruption of DNA methylation dynamics at enhancers in IDH-mutant AML cells. *Leukemia*. 2022;36(4):935–945.
