# Supplementary material for: Blood-based epigenetic instability linked to human aging and disease
Source: Nat Commun. 2026 Feb 14;17:2754. doi: 10.1038/s41467-026-69430-z (PMC13018287; doi:10.1038/s41467-026-69430-z)
Supplement: Supplementary file 2 — Description of Additional Supplementary Files [file 41467_2026_69430_MOESM2_ESM.pdf]

Description of Additional Supplementary Files

File Name: Supplementary Data 1
Description: Summary of all publicly available datasets used in this study, including dataset identifiers, associated publications, relevant sample details, and their corresponding associations with figures presented in the manuscript.

File Name: Supplementary Data 2
Description: Information on the 31,744 ESLs including: probe IDs, GRCh38 genomic coordinates, mean and standard deviation of β-values in the discovery cohort, recurrence levels, lineage enrichment annotations, CpG island association, gene association, and gene-disease associations.

File Name: Supplementary Data 3
Description: Composition of the pan-blood cancer cohort (n = 3,019) used to compute ESL recurrence.

File Name: Supplementary Data 4
Description: Cox proportional hazards regression analyses in the Framingham and cardiogenic shock cohorts with correction for 12 different immune cell fractions.

File Name: Supplementary Data 5
Description: Parameters of the top 236 age-related genes identified in the gene expression analysis, including promoter CpG island genomic coordinates and correlation statistics.

File Name: Supplementary Data 6
Description: Gene set enrichment results from g:Profiler for the 236 age-associated genes.

File Name: Supplementary Data 7
Description: Effect of 5mC on binding of transcription factors with ESLs in their binding motifs, as determined by Yin et al., 2017.
