# Supplementary material for: Blood-based epigenetic instability linked to human aging and disease
Source: Nat Commun. 2026 Feb 14;17:2754. doi: 10.1038/s41467-026-69430-z (PMC13018287; doi:10.1038/s41467-026-69430-z)
Supplement: Supplementary file 10 — Reporting Summary [file 41467_2026_69430_MOESM10_ESM.pdf]

nature portfolio

Corresponding author(s): Sagi Abelson

Last updated by author(s): Jan 6, 2026

Reporting Summary

Nature Portfolio wishes to improve the reproducibility of the work that we publish. This form provides structure for consistency and transparency in reporting. For further information on Nature Portfolio policies, see our Editorial Policies and the Editorial Policy Checklist.

Statistics

For all statistical analyses, confirm that the following items are present in the figure legend, table legend, main text, or Methods section.

n/a	Confirmed	
☐	☒	The exact sample size (n) for each experimental group/condition, given as a discrete number and unit of measurement
☐	☒	A statement on whether measurements were taken from distinct samples or whether the same sample was measured repeatedly
☐	☒	The statistical test(s) used AND whether they are one- or two-sided<br>*Only common tests should be described solely by name; describe more complex techniques in the Methods section.*
☐	☒	A description of all covariates tested
☐	☒	A description of any assumptions or corrections, such as tests of normality and adjustment for multiple comparisons
☐	☒	A full description of the statistical parameters including central tendency (e.g. means) or other basic estimates (e.g. regression coefficient) AND variation (e.g. standard deviation) or associated estimates of uncertainty (e.g. confidence intervals)
☐	☒	For null hypothesis testing, the test statistic (e.g. F, t, r) with confidence intervals, effect sizes, degrees of freedom and P value noted<br>*Give P values as exact values whenever suitable.*
☒	☐	For Bayesian analysis, information on the choice of priors and Markov chain Monte Carlo settings
☐	☒	For hierarchical and complex designs, identification of the appropriate level for tests and full reporting of outcomes
☐	☒	Estimates of effect sizes (e.g. Cohen's d, Pearson's r), indicating how they were calculated

Our web collection on statistics for biologists contains articles on many of the points above.

Software and code

Policy information about availability of computer code

Data collection	No software was used for data collection in this study.
Data analysis	Analyses were performed in Python (v. 3.11; https://www.python.org/) or R (v. 4.2.2; https://www.r-project.org/) using custom scripts. The code to reproduce all analyses in this study is available at https://github.com/abelson-lab/DNA-Methylation-Instability.

For manuscripts utilizing custom algorithms or software that are central to the research but not yet described in published literature, software must be made available to editors and reviewers. We strongly encourage code deposition in a community repository (e.g. GitHub). See the Nature Portfolio guidelines for submitting code & software for further information.

Data

Policy information about availability of data

All manuscripts must include a data availability statement. This statement should provide the following information, where applicable:

- Accession codes, unique identifiers, or web links for publicly available datasets
- A description of any restrictions on data availability
- For clinical datasets or third party data, please ensure that the statement adheres to our policy

The sources of publicly available datasets used in this study are listed in Supplementary Data 1. Newly generated methylation data of longitudinal AML samples, cardiogenic shock patients, MDS patients, and CML patients have been deposited in the GEO database under accession numbers GSE315367, GSE315366, and GEO315451. Source data are provided with this paper.

Research involving human participants, their data, or biological material

Policy information about studies with human participants or human data. See also policy information about sex, gender (identity/presentation), and sexual orientation and race, ethnicity and racism.

Reporting on sex and gender	Any CpG sites located on sex chromosomes were not considered in this study so as to avoid any sex-specific associations. Perturbation of ESLs was shown for separately for males and females with chronic lymphocytic leukemia to demonstrate this phenomenon occurs independently of sex. Biological sex was used as a covariate in Cox proportional hazards regression analyses of the Framingham Heart Study cohort.
Reporting on race, ethnicity, or other socially relevant groupings	In the analysis of ESL perturbation, three control cohorts of different ethnicities were used (Chinese descent, African descent, and unknown), ensuring that ESL stability was not specific to those of European descent (discovery cohort). These categorizations for these publicly available datasets were reported by the original authors.
Population characteristics	The characteristics of publicly available cohort data analyzed in this study can be found in their respective publications, all of which are detailed in Supplementary Data 1. The patients newly analyzed in this study are as follows:<br><br>- 4 acute myeloid leukemia patients (age 18-63) sampled at four time points (diagnosis, remission 1, remission 2, relapse)<br>- 10 acute myeloid leukemia patients (age 34-70) sampled at three time points (diagnosis, remission 1, remission 2)<br>- 57 myelodysplastic syndrome patients (age 31-83) sampled at diagnosis<br>- 69 chronic myeloid leukemia patients (age 19-80) sampled at diagnosis<br>- 64 cardiogenic shock patients (age 18-93, 29/64 with clonal hematopoiesis diagnosis)
Recruitment	For AML patients, samples were obtained from the Leukemia Tissue Bank at the Princess Margaret Cancer Centre, University Health Network.<br>For cardiogenic shock patients, we included all patients aged 18 years or older with consent and biospecimens in the Peter Munk Cardiac Centre Cardiovascular Biobank.
Ethics oversight	AML patients samples were collected in accordance with procedures approved by the Research Ethics Board of the University Health Network (REB #01-0573). Blood biospecimens from cardiogenic shock patients were collected under UHN REB approval (#18-6188) from the Peter Munk Cardiac Centre Cardiovascular Biobank. Written informed consent was obtained from all patients in accordance with the Declaration of Helsinki. University of Toronto REB approval was obtained for the use of datasets in secondary data analysis under Protocol #00041924.

Note that full information on the approval of the study protocol must also be provided in the manuscript.

Field-specific reporting

Please select the one below that is the best fit for your research. If you are not sure, read the appropriate sections before making your selection.

☒ Life sciences ☐ Behavioural & social sciences ☐ Ecological, evolutionary & environmental sciences

For a reference copy of the document with all sections, see nature.com/documents/nr-reporting-summary-flat.pdf

Life sciences study design

All studies must disclose on these points even when the disclosure is negative.

Sample size	Publicly available datasets were selected to maximize the number of samples available for analysis. Most analyses in this study examine hundreds to thousands of patients and reveal highly significant associations, suggesting the sample sizes are adequate.<br><br>For cardiogenic shock patients, we conducted a power analysis to determine the required sample size of a sub-cohort that would provide sufficient statistical power to detect a significant effect (see "Power Calculation" in the Methods).
Data exclusions	For analysis of clinical outcomes in the Framingham Heart Study cohort, we excluded patients who had experienced any endpoint (cardiovascular disease, coronary heart disease, congestive heart failure) prior to blood draw. These patients were not relevant for a time-to-event analysis given that they had already experienced the event.<br><br>Exclusion criteria were pre-established.
Replication	Stable methylation levels at ESLs was validated in three independent cohorts.<br>Association between commonly perturbed ESLs and chromatin openness was validated in two independent datasets.<br>Correlation between DMI levels and disease status (diagnosis, remission, relapse) was reproducible across all 4 AML patients with longitudinal bone marrow samples.<br>Correlation between DMI levels and somatic mutation allele frequencies was reproducible across all 10 AML patients with longitudinal peripheral blood samples.<br>Correlation between DMI and age was validated in four independent cohorts.
Randomization	The study design was not randomized as there were no treatments being tested on the patients analyzed.
Blinding	This study aims to retrospectively identify associations between abnormal methylation levels at specific loci and various disease states, and

thus blinding was not relevant.

Reporting for specific materials, systems and methods

We require information from authors about some types of materials, experimental systems and methods used in many studies. Here, indicate whether each material, system or method listed is relevant to your study. If you are not sure if a list item applies to your research, read the appropriate section before selecting a response.

Materials & experimental systems

n/a	Involved in the study
☒	☐ Antibodies
☒	☐ Eukaryotic cell lines
☒	☐ Palaeontology and archaeology
☒	☐ Animals and other organisms
☒	☐ Clinical data
☒	☐ Dual use research of concern
☒	☐ Plants

Methods

n/a	Involved in the study
☒	☐ ChIP-seq
☒	☐ Flow cytometry
☒	☐ MRI-based neuroimaging

Plants

Seed stocks

Report on the source of all seed stocks or other plant material used. If applicable, state the seed stock centre and catalogue number. If plant specimens were collected from the field, describe the collection location, date and sampling procedures.

Novel plant genotypes

Describe the methods by which all novel plant genotypes were produced. This includes those generated by transgenic approaches, gene editing, chemical/radiation-based mutagenesis and hybridization. For transgenic lines, describe the transformation method, the number of independent lines analyzed and the generation upon which experiments were performed. For gene-edited lines, describe the editor used, the endogenous sequence targeted for editing, the targeting guide RNA sequence (if applicable) and how the editor was applied.

Authentication

Describe any authentication procedures for each seed stock used or novel genotype generated. Describe any experiments used to assess the effect of a mutation and, where applicable, how potential secondary effects (e.g. second site T-DNA insertions, mosiacism, off-target gene editing) were examined.
